# Protocol for a cluster-randomised controlled trial evaluating the impact of a preschool-based capacity building intervention on intimate partner violence and substance misuse in Sri Lanka

**DOI:** 10.1186/s12889-018-5423-8

**Published:** 2018-05-02

**Authors:** Kamalini Lokuge, Polly Wallace, Kalini Subasinghe, Katherine Thurber, Tissa De Silva, Naomi Clarke, Dulshika Waas, Nisansala Liyanage, Udena Attygalle, Bradley Carron-Arthur, Kalyana Rodrigo, Emily Banks, Cate D’Este, Thilini Rajapakse

**Affiliations:** 1 The Australian National University National Centre for Epidemiology and Public Health, Canberra, Australia; 2 People’s Policy Institute, Colombo, Sri Lanka; 3 Department of Psychiatry, University of Sri Jayawardenapura, Colombo, Sri Lanka; 4 Colombo South Teaching Hospital, Kahibowlia Colombo, Sri Lanka; 5 Teaching Hospital Karapitiya, Galle, Sri Lanka; 6 Psychiatry Department, The Canberra Hospital, Woden, Australia; 7 The University of Newcastle, School of Medicine and Public Health, Newcastle, Australia; 8 Psychiatry Department, University of Peradeniya, Kandy, Sri Lanka

**Keywords:** Intimate partner violence, Sri Lanka, Preschool, Randomised control trial, Substance misuse

## Abstract

**Background:**

Past research has identified links between intimate partner violence (IPV) and alcohol misuse and poverty in Sri Lanka. Services that address substance misuse are amongst the few interventions shown to reduce IPV in settings similar to Sri Lanka. This paper describes the protocol for a study examining the impact of a preschool-based capacity building intervention on the prevalence of IPV and substance misuse in parents with children attending preschools, including uptake of available government services.

**Methods:**

The study is a cluster randomised controlled trial. Government-managed preschools (*n* = 34) in Galle and Colombo municipalities  will be randomly assigned to an intervention (*n* = 17) or control group (*n* = 17). Parents with children attending these preschools will be recruited to participate. The study intervention will build the capacity of selected community volunteers (parents) and preschool teachers in the provision of information and support to families affected by IPV and substance misuse. This intervention is directed at improving uptake, access and coordination of existing services. Data will be collected from all parents, and teachers in the intervention group, pre-intervention and 10 months post-intervention. The primary outcome for this study is experience of IPV amongst mothers of preschool-attending children. Secondary outcomes are substance misuse amongst fathers, measured via the locally adapted Alcohol Use Disorders Identification Test and Drug Abuse Screening Test; and awareness and uptake of services for these issues measured through locally-relevant tools. Demographic information and satisfaction with the intervention will also be assessed.

**Discussion:**

By intervening through preschools we aim to support high-risk families early enough to arrest the cycle of violence that results in children themselves becoming victims and perpetrators of such violence. The innovative project design will reach the most vulnerable sections of the community and will provide a sustainable and feasible strategy for scale-up of the intervention.

**Trial registration:**

This study is registered with the Sri Lankan Clinical Trials Registry (2017/038) and has been submitted to ClinicalTrials.gov (U.S National Institutes of Health) under the title “Randomized control trial: preschool-based training and support programs to reduce intimate partner violence (IPV) by addressing alcohol and drug misuse in young families in Sri Lanka”; Registration number: NCT03341455; Registration date: 14 November 2017.

## Background

Past research has identified that intimate partner violence (IPV) in Sri Lanka is often associated with poverty and alcohol misuse [1]. Such violence is linked to high levels of psychological distress among children [2, 3], and childhood experience of IPV is also a strong predictor of experiencing or using violence as an adult [1]. Drug misuse is an increasing problem in Sri Lanka [1] and is also likely to contribute to IPV. Ensuring that those affected by IPV or substance (drug and alcohol) misuse receive support is critical in improving long-term outcomes. Of particular importance is ensuring that this support is received early enough to disrupt the inter-generational cycle of violence that can result from childhood exposure to these risks.

Services that address alcohol misuse by perpetrators of domestic violence are amongst the few interventions shown to reduce IPV in settings similar to Sri Lanka [4, 5]. Services for addressing IPV and substance misuse in Sri Lanka are provided through both government and non-governmental agencies [6], but formal, integrated IPV and substance misuse programs do not exist. Uptake of services by those experiencing IPV is low, limited by factors including lack of awareness, poverty, stigma and limited coordination between different referral sectors. Interventions that improve the coordinated uptake of available services for IPV and substance misuse in Sri Lanka are needed and have the potential to not only improve substance use problems, but also to impact positively on prevalence of IPV.

Evidence, including from Sri Lanka, suggests that women suffering from IPV are more likely to seek support from family and community members in preference to formal support agencies [3, 7]. There is also evidence from high income settings that lay community members can be trained to effectively support those experiencing IPV to access services [8].

Preschool teachers and volunteer mothers and fathers (from the intervention group) will be trained as lay community members to support young families experiencing IPV or substance misuse. They will receive training on the impacts of parental IPV and substance misuse on children’s educational achievements, key messages to convey to families experiencing IPV or substance misuse, and practical information and resources for accessing services to assist families including contact details, referral criteria, duration of available programs, expected commitment of participant, costs of services and likely impact or benefit of accessing the available services.

Preschools offer a route through which to implement community support programs to increase awareness, knowledge and uptake of services for IPV and substance misuse, and to target such programs to families with young children. Additionally, the preschool setting provides a route for delivering preventive messages in relation to these issues. The primary aim of the study is to determine whether such preschool-based capacity building programs reduce the prevalence of IPV in preschool-attending families. Secondary aims include assessing the impact of such programs on awareness and uptake of services, and on prevalence of alcohol and drug misuse in these families.

## Methods

We hypothesise that building the capacity of selected parents and preschool teachers in the provision of information and support for accessing appropriate government services to young families attending preschools who may be affected by IPV and substance misuse will have positive effects on:

- Prevalence of IPV reported by women in the intervention group
- Prevalence of drug and alcohol misuse reported by men in the intervention group
- Awareness and uptake of available government services that address these issues  by young families

We will address the hypothesis using a randomised controlled trial (RCT) to deliver and evaluate a community-based advocacy intervention that is affordable, adaptable, effective, and community driven.

### Study design

**Fig. 1:**
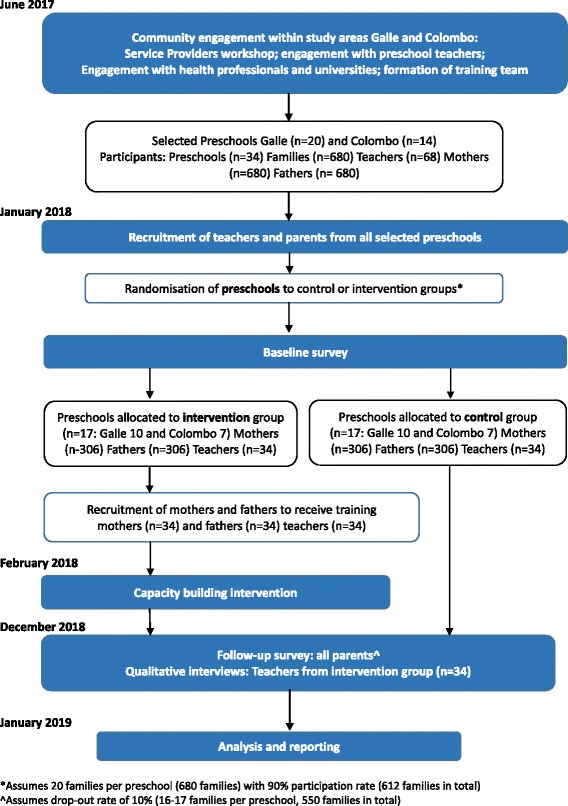
Study design and timeline

The study is a preschool-based parallel group cluster RCT that will be conducted to assess the impact of a capacity building intervention on IPV and alcohol and drug misuse related knowledge, practices and prevalence. The comparison is between preschools who receive targeted training (intervention group) and the preschools that do not (control group). Parents with children attending government-managed preschools in two study areas will be recruited to participate. Conventional RCTs, in which individuals are randomly allocated to different treatment groups, are inappropriate for interventions where the level of allocation is a cluster, based around a health facility, village or preschool as is the case in this study. Figure 1 provides an overview of the study design.

### Setting

Preschools in Sri Lanka are attended by children aged between 3 and 5 years for at least one year prior to starting school and coverage in this age group is estimated at 46% [9]. The majority of preschools are privately run. Although government-run preschools constitute only 16% of all preschools [9] they are free of charge and are generally most utilised by economically disadvantaged sections of the community.

Education is highly valued in Sri Lanka, even amongst the most socio-economically disadvantaged, and parents will invest significant resources in ensuring the educational success of their children. Based on discussions with preschool teachers and community service providers (both government and non-government) we assert that explicitly linking IPV and substance misuse to poorer educational outcomes for children will support prevention of these issues, as well as provide motivation to those parents affected by these issues to seek assistance. By ensuring information on such assistance is accessible through trusted community members, we hope to improve uptake, retention and post-treatment effectiveness of support services.

Our intervention will build the capacity of preschool teachers in providing preventive educational messaging, selected mothers in the provision of information and support for accessing services for IPV, and selected fathers in the provision of information and support for accessing services for substance misuse support services. By delivering these interventions through government preschools, we aim to deliver an intervention that targets poorer families with young children that is effective, feasible and sustainable within existing government and community resources and infrastructure.

### Target population

Our target population is families utilising government preschools in two urban areas in Sri Lanka: Galle urban municipality (Galle) and Dehiwala Mt Lavinia municipality (Colombo). The urban site in Galle covers 20 government-run preschools and Colombo covers 14. All parents of children attending these preschools will be eligible for inclusion in the study, and will be included if consenting.

### Recruitment

All parents from participating preschools will be invited to participate in the study. This invitation will be in the form of a written letter and verbal announcement made by the project intervention team. The parents who volunteer to participate will be invited to attend the school after-hours on a designated day to receive additional information and complete the baseline survey under the supervision of the data collection team. Mothers and fathers will be asked to attend on separate days. Two sessions (a weekday and a weekend session) will be offered to each group to allow participants a choice in the time at which they undertake the survey.

Participation is voluntary and any participant can leave the study at any time they wish. If a participant does leave during the duration of the intervention, any data provided by them up to that point will remain part of the study. Only de-identified data are collected. Therefore, the data collection team and data entry officer are unable to identify any participants who remain or withdraw from the study based on the information received.

In the randomly selected preschools that will receive the intervention, all mothers and fathers will be invited to volunteer to receive capacity building and training as part of the intervention. We aim to recruit two mothers and two fathers participating in initial workshops from each preschool. Those who volunteer will be interviewed by the training team (which is distinct from the data collection team) and selected based on their performance in an initial workshop. Mothers and fathers will attend separate workshops on different days. Training will be delivered to mothers and fathers separately on different days.

All teachers from participating preschools will be invited to attend the teacher training and will be included if they consent. All teachers, 10 randomly selected mothers and 10 randomly selected fathers who participated in the training, will be interviewed at the conclusion of the study.

### Randomisation

Randomisation will be at the level of the preschool stratified by study area. Half the preschools in each study area will be randomly allocated to receive the teacher and parent training intervention at the beginning of the preschool year (*n* = 17: 10 preschools in Galle and 7 in Colombo). The preschools that do not receive the intervention (*n* = 17: 10 preschools in Galle and 7 in Colombo) will act as time-concurrent controls. If the intervention is demonstrated as being effective, these control preschools will receive the intervention at the conclusion of the study.

Randomisation will be carried out by the study statistician based outside the study areas, blinded to study area and preschool, in a 1:1 ratio to intervention or control group stratified by study area and using a computer generated randomisation process. The allocation program will be archived for reproducible research compliance.

### Blinding

Allocation of interventions cannot be concealed from either training staff or study participants once implementation occurs. However, all data collectors will be blinded to the intervention status of preschools. The study will be presented as a project related to family health and wellbeing, no specific study hypotheses will be presented to participants, and data collectors will not be involved in delivering the intervention. However, parents at preschools receiving the intervention will be aware of the training activities, and may still link the intervention to survey activities.

The possibility of parents from a control group preschool changing to an intervention group preschool at different stages (pre or post intervention) of the study in order to receive the intervention, will be minimised by the intervention being implemented after preschool enrolments have been completed for the school year. Participants from control areas may also obtain information on service use through interaction with intervention area households, or through having siblings attending control and intervention area preschools. To assess the degree to which this may occur, we will collect data on participant households preschool enrolment details, residence and sources of information and utilisation of intervention-relevant services.

### Data collection

Data from parents will be collected in de-identified form through anonymous, self-administered surveys. Literacy rates in Sri Lanka are very high for both men and women, so we do not expect illiteracy to be a barrier to participation. Data collection will be supervised by staff recruited specifically for this task. These staff will receive training prior to commencement of the baseline data collection which address the contents of the survey and how to provide confidential, safe and appropriate support to participants who seek or request assistance, including for domestic violence or substance misuse, during completion of the survey. Refresher training will be provided to the same team prior to the collection of post intervention data.

All government preschools in the study areas will be involved in the following data collection activities:

- A baseline survey of mothers and fathers at the beginning of the preschool year (January 2018)
- A follow up survey of all mothers and fathers at the end of the preschool year (December 2018).

Preschools receiving the intervention will participate in the following additional post intervention data collection activities:

- Qualitative interviews with key participants including teachers, community members, community development staff and other government sector service providers to determine their satisfaction with the intervention, and any challenges or barriers to its implementation.

### Capacity building intervention

We aim to implement a community-based support program delivered by preschool teachers and volunteer parents that will increase awareness, knowledge and uptake of available services for IPV and substance misuse, and of the link between these issues and poorer education outcomes in children. Through this, we aim to decrease the prevalence of IPV and substance misuse.

The intervention design was developed prior to delivery of the baseline survey through discussions and  workshops attended by government service providers, including welfare, health, policing, substance misuse and education providers. These workshops demonstrated the availability of a comprehensive range of services across IPV, substance misuse and other social services. A key finding from these workshops was the need for improved links between such services and the community, in particular the need to more effectively engage men. Another important finding was that promoting children’s education was considered a promising route through which to encourage positive behaviour change.

The following specific activities and interventions will be implemented in the intervention preschools: Capacity building, training and support to selected mothers on the provision of safe, confidential and relevant community-based referral and support services to women affected by IPV; Capacity building, training and support to selected frathers on the provision of safe, confidential and relevant community-based referral and support to men seeking support for substance misuse problems; Capacity building and on-the-job training and support to preschool teachers on provision of IPV and substance misuse prevention educational messages (including the links between these issues and poor child development [1]), and referral pathways to sevices for these issues. The training intervention will be delivered after completion of the baseline survey and randomisation (see Fig. 1).  Training for mothers will focus on what IPV services are available and how to access them. These trained community members will work with those mothers experiencing IPV to: define which health, safety, protection and justice goals they are seeking in accessing services; ensure they are aware of how to access services relevant to these goals; and support them in the often drawn-out process of achieving these goals. The value of such programs in supporting those affected by IPV suggests they may also be an effective means of supporting uptake of substance misuse support services for fathers in the same way.

Training is essential to ensure these community members have the skills and knowledge to provide safe, non-judgmental and confidential information and ongoing support. Training and resource materials will be developed through workshops with local service providers, with input from technical experts including social workers, psychiatrists and psychologists experienced in addressing IPV and substance misuse in Sri Lanka and internationally. These will cover information on available services for IPV and substance misuse including contact details, referral criteria, duration of available programs, expected commitment of participant, costs of services and likely impact of benefit outcomes of accessing services.

The training team, responsible for delivering the intervention, will include local experts involved in developing the training materials who have expertise in providing services to families and individuals affected by substance misuse and IPV. Training will focus on non-judgemental support and will be delivered in the local language of the community on separate dates to, and after completion of, baseline surveys.

Materials developed for this study will build on and adapt existing resources developed locally and internationally shown to have value in multiple settings, including those with limited resources. Such training interventions include those utilised in community-based advocacy and support services [8], and community mobilization interventions developed to prevent violence against women [1, 2].

Through the training of selected fathers in the preschool community a similar model will be applied to ensure that those needing support for substance misuse are aware of available services and how to access them, and complete substance abuse programs.

Discussions with stakeholders identified the need for preparatory community outreach activities in order to familiarise the community with the project team. These activities were considered necessary to effectively engage the study communities, in particular men, to support good uptake of the intervention. These community engagement activities will commence in both study areas prior to implementing the intervention and will target the community as a whole (regardless of their status as intervention or control preschool areas). Activities will be developed based on the experience of service providers working for several years in these areas and include general health, sanitation and education related information and resources, delivered at community meetings by members of the project team.

The impact of community-based support and advocacy to address IPV and substance misuse is dependent on how effective such interventions are in mobilising existing services, in particular government health, policing, justice and welfare services. In each study area designated government Community Development Officers (CDOs) manages all of the government preschools in that area. The role of the CDOs include regular meetings with preschools and oversight of local-level livelihood programs and other welfare services. By including the CDOs in this capacity building intervention, we will strengthen the link between preschools and government service providers who are well placed to ensure access to longer-term support for families.

Our intervention is broadly structured to address the different stages of behaviour change and decisional balance [10]. Linking positive behaviour change to the educational success of preschool children is designed to move individuals from the stages of pre-contemplation to contemplation by influencing the way individuals assess the pros and cons of particular behaviours. Providing trusted sources of peer-support and information is designed to support individuals to move through the stages of preparation, action and maintenance of behaviour change by supporting increased self-efficacy.

In developing, designing and implementing the intervention, our work was underpinned by a framework of community engagement based on the following principles: a shared vision; mutual respect; awareness; shared responsibility; building capacity; improved coordination; inclusiveness; appropriate timeframe; sustainability; and integrity [11].

### Outcome measures

Of interest are differences at 10 months post-intervention between families in control and intervention groups in:

- the primary outcome: experience of intimate partner violence by mothers in this group measured using instruments developed and validated across multiple countries for the World Health Organization (WHO) multi-country survey of violence against women [12] ^.^and
- secondary outcomes of interest:
  - ◦ Prevalence of substance misuse in fathers in this group measured via the locally adapted Alcohol Use Disorders Identification Test (AUDIT) [13] and the Drug and Alcohol Screening Test (DAST) [14]; and
  - ◦ Awareness and uptake of services for IPV and substance misuse measured through locally-relevant tools developed during initial project workshops.

Process measures that will be assessed through interviews in intervention areas include:

- Teachers’ and volunteer mothers’ knowledge and skills in regards to the initial assessment, management and referral of families affected by IPV.
- Volunteer fathers’ knowledge and skills in the initial assessment, management and referral of families affected by substance misuse.
- Community and service provider satisfaction with the intervention, assessed through key informant interviews with community development staff and government service providers (health, welfare, police etc)

#### Other factors to be assessed

Factors identified as associated with IPV and substance misuse in previous research include socio-economic status, educational status, age, marital status and mental health. Data on these factors will be collected, including on mothers’ and fathers’ mental health and depression using a locally developed and validated screening tool for depression (the Peradeniya Depression Scale) [15]. We also postulate that family and community functioning, including measures of social cohesion and inclusion, may be associated with the outcomes of interest. These will be assessed through locally adapted indicators and rating scales measuring family and children’s resilience [16].

#### Sample size

The sample size was calculated to demonstrate a significant difference in the proportion of mothers of preschool children reporting exposure to IPV following intervention. We plan to recruit at least 34 preschools, with 20 families per preschool, for a total of 680 potential families (which equates to 340 families in the intervention group and 340 in the control group who may be approached to participate in this study. We anticipate minimal loss to drop out due to the educational focus on children this study promotes.

We assumed a participation rate of 90%, based on past research which found a consent rate in this population for domestic-violence related surveys of 97% [3]. We therefore expect a sample size of approximately 612 families, or 306 per group. Based on a design effect due to correlation of outcomes within preschools of 1.5 (which equates to an intra-class correlation coefficient of 0.025), 80% power, a 5% significance level and a pre-intervention 12 month prevalence of IPV of 40% [3] we will be able to detect a difference in post intervention 12 month prevalence of IPV between intervention and control groups of 15%.

There are no intervention studies in Sri Lanka or that region that examine the effects of community-based interventions. However, an extensive community-based intervention (SASA!) in Uganda, Africa reported a 53% reduction in physical IPV reported by women in the intervention group [17]. Therefore, we consider it justifiable to expect an intervention effect of at least 15%.

#### Data management and quality assurance

Data will be entered into password-protected data management software by data entry staff during both survey rounds (baseline and follow-up) and will be assessed for quality using standard queries in the software package. Any issues in data quality will be identified and addressed through discussions between the data collection team and project supervisors in the field. Hardcopies of completed surveys will be stored securely for five years at the study co-ordinating site. Data will be accessible to the study investigators as per the conditions of the ethical approvals. Any additional individuals within or external to the study requesting access to the study data will be required to apply to the ethical review committees.

#### Data analysis

The main analysis will be by intention-to-treat. The primary analysis will be a regression model of IPV experienced by mothers within the past 10 months in the intervention group, adjusted for other potential confounders, and correlation of outcomes within preschools using a Generalised Estimating Equations approach. Primary analysis will be a complete case analysis.

Similar analysis will be conducted for secondary outcomes including prevalence of substance misuse in fathers and awareness and uptake of services for IPV and substance misuse. Secondary analysis will use multiple imputation. Sensitivity analysis will be conducted through secondary adjusted analysis accounting for potential confounders. Confounders specified a priori as being potentially associated with the outcome include age, mental health status and socio-economic status.

#### Dissemination of study findings

The results of the study will be provided as a written report to key stakeholders including local government staff and donors. This report will also be presented and discussed with relevant national government agencies by the local study team. A summarised form of the report will be provided to community staff and preschool teachers for discussion and feedback with parents. Support will be provided by the study team to facilitate such discussions. The study results will also be reported in peer-reviewed publications.

#### Ethical considerations

Parents will be invited to participate in the study as part of a family well-being initiative. The full nature of the study, in terms of the emphasis on IPV, will not be divulged to anyone except the mothers participating and those involved in the support intervention, as recommended by the World Health Organisation guidelines for research into violence against women [18]. Parents will not be aware of the IPV (for mothers) or substance misuse (for fathers) content of the study until they attend the school as per their invitation. Mothers and fathers will attend the school at different times and be issued with different participant information sheets that will contain details of IPV (for mothers) and alcohol and drug support services (for fathers) in the local area. Also listed will be the contact details for the Ethical Review Committees and local study coordinators.

Individual consent will be obtained from all participants before any information is collected from them. Information will be collected from parents via anonymous, self-administered surveys. Mothers and fathers will provide different data by completing different surveys (one for all mothers and one for all fathers). For the mothers, a second stage of written consent will occur within the questionnaire, after full disclosure of the nature of the questions on family violence. If women do not wish to continue, they can submit their questionnaire without signing the second stage of consent and without completing the sections on family violence.

Researchers will not be able to link data to a particular individual within any preschool because identifiable data (including name) are not collected. This ensures confidentiality of response. During completion of the surveys by mothers and fathers, a trained survey team member will be available to assist (in a private space) if help is requested either to complete the survey or to access services.

The Institutional Ethical Review Committee, University of Peradeniya, Sri Lanka (2017/EC/17) and the Human Research Ethics Committee, Australian National University (2017/066), have granted approval for the study. Oversight of the study will be through regular reporting to these ethical review committees.

## Discussion

It is not possible to blind participants to the intervention and, as outcomes are self-reported, there is the potential for bias on the part of participants, particularly if participants are able to link the intervention to the surveys through which outcomes will be quantified. To address this, the data collection team will be completely separate from the training team who deliver the intervention, and participants will not be informed of a link between these two activities. The data collection team will also be blinded to whether participants are from intervention or control preschools.

The preschool setting limits the time available to evaluate the intervention. Children usually attend preschool for one year. Our follow up surveys must be completed by the end of the school year (December 2018) otherwise participants will be lost to follow up as no contact details are collected. This provides us with a maximum of ten months to evaluate our intervention. Past research has shown that positive effects from community interventions may not be reported until later follow up of 24 months [8].

No identifying data will be collected. Therefore there is no possibility of following up any participants who drop out of training or do not complete either survey.

Our research is limited to urban areas and may not be generalised to rural areas. As a separate component of this study, we are also piloting the program in three rural preschools. Levels of service provision are generally lower in such areas and, through this pilot, we aim to determine the adaptations required to successfully implement a similar program in rural areas of Sri Lanka. Qualitative data will be collected in the rural preschools to determine the feasibility, acceptability and adaptations necessary for implementation of this intervention in rural areas. Data will be collected through interviews with all teachers and mothers and fathers who participated from each of the three preschools.

## Conclusion

This study will develop, implement and evaluate a community capacity building intervention aimed at reducing IPV and alcohol and drug misuse in communities in Sri Lanka. The innovative method of implementation, delivering this training to parents of children utilising preschools and preschool teachers will reach the most vulnerable sections of the community and will provide a sustainable and feasible strategy for scale up of the project. By intervening through these preschools we aim to build capacity within the community to  identify and support high-risk families early enough to arrest the cycle of violence that results in children themselves becoming victims and perpetrators of such violence.

This research will address important gaps in literature documented by other researchers [3] by robust evaluation through repeated cross-sectional surveys in the target population to assess the effectiveness of the intervention. Preschool communities have not previously been target populations when addressing IPV in Sri Lanka. Results will add to the evidence base for future interventions in Sri Lanka and have potential to contribute to national policy.

## Abbreviations

CDO: Community Development Officer
IPV: Intimate Partner Violence
RCT: Randomised Controlled Trial
WHO: World Health Organization
